# O.5.3-5 Existing structures for physical activity promotion: the role of resources, political support, intersectoral collaboration, and participation in six pilot communities - a comparative case study-

**DOI:** 10.1093/eurpub/ckad133.256

**Published:** 2023-09-11

**Authors:** Natalie Helsper, Lea Dippon, Simone Kohler, Leonie Birkholz, Philipp Weber, Klaus Pfeifer, Alfred Ruetten, Jana Semrau

**Affiliations:** Friedrich-Alexander-Universität Erlangen-Nürnberg, Germany; natalie.helsper@fau.de; Friedrich-Alexander-Universität Erlangen-Nürnberg, Germany; natalie.helsper@fau.de; Friedrich-Alexander-Universität Erlangen-Nürnberg, Germany; natalie.helsper@fau.de; Friedrich-Alexander-Universität Erlangen-Nürnberg, Germany; natalie.helsper@fau.de; Friedrich-Alexander-Universität Erlangen-Nürnberg, Germany; natalie.helsper@fau.de; Friedrich-Alexander-Universität Erlangen-Nürnberg, Germany; natalie.helsper@fau.de; Friedrich-Alexander-Universität Erlangen-Nürnberg, Germany; natalie.helsper@fau.de; Friedrich-Alexander-Universität Erlangen-Nürnberg, Germany; natalie.helsper@fau.de

## Abstract

**Purpose:**

The German Recommendations for Physical Activity (PA) and PA Promotion recommend community-based PA promotion (c-PAP) with focus on health equity. As communities are complex systems with dynamic relationships and interactions, a deeper understanding of the community’s structures is crucial for implementation. Therefore, the aim of this study is to analyse the initial structures in six diverse pilot communities (PC).

**Methods:**

A framework for c-PAP was co-produced and implemented in six PC, which varied in terms of type (urban, rural), region, readiness, and socioeconomic deprivation.

Using a comparative case study design, we conducted qualitative interviews with key stakeholders from each PC including political stakeholders, experts, representatives of the administration, “door-openers” with contact to people with social disadvantages, and community facilitators. The development of a semi-structured interview guideline was based on the co-produced nine KC. With a qualitative content analysis, we compared similarities and differences in the initial structures of the six PC along the following four KC: “resources”, “political support”, “intersectoral collaboration”, and “participation”.

**Results:**

32 interviews revealed differences in the four analysed KC according to the community type, the socioeconomic deprivation as well as the readiness. “Intersectoral collaboration” was stronger in most urban-PC. The KC “participation” varied according to previous experiences in participatory approaches. Although urban PC have had more experiences, these were not necessarily positive and sometimes lead to reservation about new approaches. “Political support” seemed to be higher in rural-PC. Available resources differed according to the socioeconomic deprivation and rural-PC in general had fewer financial and personnel resources at disposal.

**Conclusions:**

A thorough analysis of the initial community structures will contribute to a better understanding of how the structures influence process, output, and outcome of community-based PA promotion. Finally, it will support an enhanced exploration of the impact regarding sustainable and equitable structural change for PA promotion at community level.

**Funding Source:**

This work was supported by the Federal Centre of Health Education (BZgA) on behalf of and with funds from the statutory health insurances according to § 20a SGB V in the context of the GKV Alliance for Health (www.gkv-buendnis.de).

